# COVID-19 and the impact of physical activity on persistent symptoms

**DOI:** 10.3389/fspor.2025.1560023

**Published:** 2025-04-24

**Authors:** Lauren E. Opielinski, Toni D. Uhrich, Michael H. Haischer, Rachel N. Beilfuss, Lindsey M. Mirkes Clark, Kamryn M. Kroner, Rachel E. Bollaert, Michael J. Danduran, Linda B. Piacentine, Marie Hoeger Bement, Paula E. Papanek, Sandra K. Hunter

**Affiliations:** ^1^ Exercise Science Program, Marquette University, Milwaukee, WI, United States; ^2^ Athletic and Human Performance Research Center, Marquette University, Milwaukee, WI, United States; ^3^ Department of Physical Therapy, Marquette University, Milwaukee, WI, United States; ^4^ College of Nursing, Marquette University, Milwaukee, WI, United States

**Keywords:** COVID-19, physical activity, persistent symptoms, accelerometer, long COVID, healthy control, fatigue

## Abstract

**Introduction:**

Physical activity is protective against chronic disease but whether activity is associated with persistent symptoms in non-hospitalized coronavirus disease 2019 (COVID-19) survivors is unknown. The purpose of the study was to determine the impact of the COVID-19 pandemic on physical activity levels and the influence of physical activity on acute COVID-19 and long COVID symptoms in non-hospitalized COVID-19 survivors.

**Methods:**

In total, 64 non-hospitalized COVID-19 survivors (45 female participants, 40 ± 18 years) were assessed for activity levels, body composition, and symptoms of COVID-19 8.5 ± 4.7 months post-infection and categorized into two groups: (1) persistent symptoms and (2) no symptoms at the time of testing. Furthermore, 43 of the 64 participants (28 female participants, 46 ± 18 years) completed a follow-up questionnaire online 51.0 ± 39.7 months (4.25 years) post-infection. A subset of 22 COVID-19 survivors (16 female participants, 35 ± 16 years) were matched for age, sex, and body mass index with healthy controls. Physical activity was quantified using (1) self-reported questionnaire (International Physical Activity Questionnaire; IPAQ-SF) at three time periods; prior to COVID-19 infection, at the time of laboratory testing (8.5 ± 4.7 months after infection), and during an online follow-up (51.0 ± 39.7 months, i.e., 4.25 years after infection); and (2) 7 days of wearing an ActiGraph accelerometer following laboratory testing.

**Results:**

Physical activity (IPAQ-SF) declined in COVID-19 survivors from pre-COVID-19 infection to 8.5 ± 4.7 months after infection [3,656 vs. 2,656 metabolic equivalent of task (MET) min/week, 27% decrease, *p* < 0.001, *n* = 64] and rebounded to levels similar to pre-COVID-19 infection at 4.25 years after infection (*p* = 0.068, *n* = 43). Activity levels quantified with accelerometry did not differ between COVID-19 survivors and controls. However, COVID-19 survivors who reported persistent symptoms 8.5 months after infection (*n* = 29) engaged in less moderate-vigorous physical activity and steps/day than those without persistent symptoms (*n* = 27) (37 vs. 49 MET min/day, *p* = 0.014 and 7,915 vs. 9,540 steps/day, *p* = 0.014)*.*

**Discussion:**

Both COVID-19 survivors and matched controls reported reductions in physical activity indicating that lower levels of activity were likely due to the pandemic rather than COVID-19 infection alone. However, those who were most affected by COVID-19 infection with persistent symptoms had the greatest reductions in physical activity, even at ∼8 months and ∼4 years post-infection.

## 1 Introduction

Coronavirus disease 2019 (COVID-19) is caused by the virus SARS-CoV-2 and its rapid spread caused a global pandemic declared in March 2020, resulting in 7 million deaths globally and ∼1 million in the United States by 2024 (1, 2). Acute infections range from mild symptoms to severe illness requiring hospitalization, including fever or chills, cough, shortness of breath, fatigue, muscle or body aches, headache, new loss of taste or smell, and sore throat (3, 4). Most COVID-19 cases (80%–90%) are less severe and do not typically require hospitalization (5).

Some symptoms from the COVID-19 infection, even in non-hospitalized patients, are persistent and develop into the chronic condition long COVID (also known as long haul COVID, post-COVID conditions, post-acute sequelae of COVID-19, post-acute COVID-19 syndrome, etc.) (6). Long COVID is defined as symptoms for at least 3 months post-infection, with some symptoms that emerge, persist, resolve, and then reemerge (7, 8). Common long COVID symptoms include fatigue, post-exertional malaise in response to physical or mental effort, shortness of breath, heart palpitations, difficulty thinking or concentrating (brain fog), and an overall decrease in quality of life (9–11). The percentage of American adults reporting long COVID symptoms was 6.9% in 2022 and grew to 17.9% in 2024 (12, 13). The risk of long COVID is greatest in people with severe acute illness, those who were infected multiple times, and those who are unvaccinated and become infected. In addition, the risk is heightened in women, Hispanic or Latino individuals, older adults (>65 years), and people with underlying health conditions (9, 14–16).

Physical activity (PA) is effective in improving health and longevity and protects against chronic disease (e.g., cardiovascular disease, diabetes, cancer, hypertension, obesity, depression, and osteoporosis) (17, 18). Additional benefits of PA include moderation of the immune system and protection against acute diseases, including reduced incidence of viral infections, intensity of symptoms, and mortality (19–23). Regular participation in PA may influence the risk of acute infection of COVID-19 and the development of persistent symptoms with long COVID, although there is minimal information on the association between activity levels and COVID-19 infection and long COVID symptoms. A confounder to understanding the impact of activity levels on acute COVID-19 and long COVID symptoms is the isolation effects due to the pandemic on population activity levels, which declined markedly during the initial lockdowns (24–28).

A person's level of physical activity and sedentary behavior prior to the pandemic, however, appears to be predictive of the most severe effects of acute COVID-19 infection. From large datasets of medical records of people who had been hospitalized with COVID-19, it was shown that individuals who reported being consistently inactive had higher rates of ICU admission and death due to COVID-19 (20, 29). In addition, even people who participated in some PA but did not meet the recommended guidelines of 150 min of moderate-intensity physical activity per week had more positive outcomes than no activity, indicating that some PA is beneficial (29, 30). Inactivity was the strongest risk factor for hospitalization due to COVID-19, suggesting that PA is one of the most important protective therapies against acute COVID-19 infection and symptoms (21, 31, 32). Little is known about the influence of PA in those who experienced less severe infections and who did not require hospitalization.

Furthermore, the impact of PA on symptom recovery in people who have suffered long COVID is not understood. Recent studies report that PA in people with long COVID can improve persistent effects or in some worsen persistent symptoms (33–35). These mixed findings may contribute to reduced engagement and adherence to PA guidelines in individuals experiencing long COVID who may be limited by symptoms and even symptom exacerbations, including fatigue and post-exertional malaise (5, 36–38). Research focusing on longitudinal data from the acute phase throughout the potential development of long COVID is limited.

The primary aim of this study was to determine the impact of the COVID-19 pandemic on physical activity levels and the influence of physical activity on acute and long COVID symptoms in female and male COVID-19 survivors (COV) who had not been hospitalized. We hypothesized that (1) PA levels would decrease following acute COVID-19 infection in non-hospitalized COVID-19 survivors and be restored when reported years following the COVID-19 pandemic, (2) PA levels would be lower in non-hospitalized COVID-19 survivors than healthy matched controls, and (3) COVID-19 survivors with higher PA levels prior to COVID-19 infection would report the lowest number of persistent symptoms with long COVID.

## 2 Materials and methods

### 2.1 Study overview

In total, 86 participants visited the laboratory for a single session followed by 7 days of physical activity monitoring as part of a larger, single-site cross-sectional study at the Athletic and Human Performance Research Center, Marquette University, Milwaukee, WI, USA, between November 2020 and November 2022. Laboratory assessments included resting heart rate and blood pressure, anthropometrics [height, weight, and body composition from a dual-energy x-ray absorptiometry scan (DXA)], a COVID-19 history questionnaire, a physical activity questionnaire, and instruction on the 7-day accelerometry. To understand the influence of COVID-19 on PA at this session, both a self-report questionnaire on PA levels and data collection of measured PA from an activity monitor were completed. The self-report questionnaire in COVID-19 survivors was captured for two time points at this session with (1) recall prior to COVID-19 infection and (2) 8.5 ± 4.7 months after infection. In addition, PA measured via accelerometry was compared between COVID-19 survivors and control-matched participants following laboratory testing. In total, 43 COVID-19 survivors went on to complete an online follow-up survey administered via Qualtrics (Qualtrics, UT, USA) between May and June 2024 that included questionnaires on COVID-19 symptoms and PA. Self-report PA was captured for an additional third time point, i.e., 51.0 ± 39.7 months or 4.25 years after infection, during this follow-up. These three time points of self-report PA allowed for a longitudinal study design in COVID-19 survivors to understand the influence of COVID-19 on PA over a long period of time.

### 2.2 Participants

Non-hospitalized COVID-19 survivors (*n* = 64, 19–77 years, 45 female and 19 male participants) and healthy controls (*n* = 22, 21–66 years, 16 female and 6 male participants) volunteered to participate in the study. Each participant provided written informed consent prior to participation, and the protocol was approved by the Marquette University Institutional Review Board (HR-3661) in accordance with the Declaration of Helsinki for human subject research. Participants were convenience sampled from Milwaukee, WI, USA, and surrounding areas. Recruitment strategies included printed flyers, emails, and online advertisements via social media on Marquette University's campus and alumni networks and a local news channel. All interested participants were screened online prior to being invited to participate in the study using the Physical Activity Readiness Questionnaire (PAR-Q+) that assesses contraindications to exercise (39–42). Exclusion criteria included asymptomatic acute COVID-19 infection, pregnancy, and any condition or disease that would preclude the individual from the ability to perform the various tests and measures involved in the study (e.g., myocardial infarction in the last 12 months, pulmonary embolism, musculoskeletal issues, fibromyalgia, or active cancer). Participant groups included both COVID-19 survivors and healthy controls based on the following inclusion criteria:

1) *COVID-19 survivors (COV):* self-reported positive test or diagnosis of COVID-19 at least 2 months prior to the laboratory session date and not hospitalized for their COVID-19 infection.
2) *Non-COVID healthy controls (CON):* self-reported never experiencing a previous positive test, diagnosis, or symptoms of COVID-19.

The CON participants were recruited around the same time during the pandemic (within months) and were matched for age, sex, and body mass index (BMI). All participants included in the study were screened for COVID-19 symptoms and confirmed to be COVID-19 negative via a nasopharyngeal swab or saliva test on the day of the laboratory session.

### 2.3 Baseline measures

At the start of the laboratory session, resting blood pressure and heart rate were collected using an automatic blood pressure cuff (Omron Healthcare HEM-907XL, Kyoto, Japan). Height was recorded via a stadiometer (Seca, Hamburg, Germany), weight was recorded using the basic scale function of a multifrequency quadripolar bioelectrical impedance analysis scale (Tanita MC780-U, IL, USA), and body fat percentage was quantified from a DXA machine (Hologic Horizon A, MA, USA). BMI (kg/m^2^) was calculated, and BMI categories were utilized (underweight < 18.5 kg/m^2^, normal weight 18.5–24.9 kg/m^2^, overweight 25–29.9 kg/m^2^, and obese ≥ 30 kg/m^2^).

### 2.4 COVID-19 history questionnaire

All participants completed a questionnaire asking about sex, age, race, ethnicity, and COVID-19-specific vaccination history, and for the COV participants only, information about COVID-19 symptoms. At the laboratory session, the COV participants were asked to recall symptoms experienced during their first acute infection of COVID-19 via a 22-item symptom list (Supplementary Table 1) that included the most common symptoms identified by the Centers for Disease Control and Prevention. In addition, the COV participants were asked a yes/no question if they felt as though they had returned to their pre-COVID-19 state of health, and if not, they were surveyed about any new or persistent symptoms from the symptom list. The same questionnaire was included in the follow-up online survey for the COV participants (May–June 2024), including information on any new vaccinations and any new or persistent symptoms if applicable.

### 2.5 Physical activity questionnaire

PA levels were quantified using the International Physical Activity Questionnaire—Short Form (IPAQ-SF) (43). The IPAQ-SF is a self-reported seven-item questionnaire that asked about the previous 7 days and provided data on the different intensities of PA (i.e., vigorous, moderate, and walking) with appropriate examples of each and sitting time that people do as part of their daily lives. Self-reported minutes per week spent in each intensity (sitting is reported in minutes per day) were reported and contributed to the estimation of total PA in metabolic equivalent of task (MET) min week (total METs) using the IPAQ-SF. Participants filled out the IPAQ-SF for three time points: (1) retrospective recall of their PA prior to COVID-19 infection during an average week (COV only) administered at the laboratory session (25, 44), (2) previous 7 days of activity from the date (COV and CON) of the laboratory session, and (3) online follow-up survey in reference to their previous 7 days of activity from that date (COV only). Duration values from the IPAQ-SF were limited to a maximum of 180 min/day to reduce outliers according to guidelines (45, 46). For total METs and sitting time, the medians with interquartile ranges were reported according to guidelines (46).

### 2.6 Accelerometry to measure physical activity

PA was also quantified with a wearable device (ActiGraph GT3X+, FL, USA) that was distributed to all the participants (COV and CON) at the conclusion of the laboratory session. The participants were instructed to wear the device on the provided belt over the non-dominant hip for 7 days minus sleeping times (including naps) and water exposure (e.g., bathing, swimming). Upon completion of the 7-day wear, the device was returned to the laboratory for analysis. Participants were also provided with an activity log to aid in the validation and analysis of the data. Accelerometer wear time data were checked against the participant-recorded wear times from the log sheet, and only valid days (>10 h of wear time per day) were included in the analysis. Analysis was completed in ActiLife software (version 6.13.4) for average minutes per day completed for sedentary (≤99 activity counts/min), light (100–1,951 activity counts/min), and moderate-vigorous physical activity (MVPA) (≥1,952 activity counts/min) (47) and average step count per day.

### 2.7 Statistical analysis

Prior to analysis, variables were screened for normality using the Shapiro–Wilk test to assess data for normal distribution in addition to visual inspection of Q-Q plots and evaluation of skewness and kurtosis. In addition, variables were screened for linearity using Levene's test to assess the data to ensure homogeneity of variances. To verify the accurate matching of pairs between COV and CON participants, paired samples *t*-tests or Wilcoxon signed-rank tests were performed as appropriate on the matching criteria of age and BMI in addition to other variables from the participant demographics. Further analysis into the differences between these pairs for PA variables from the measured accelerometer and IPAQ-SF was completed accordingly. Comparisons between groups based on sex for participant descriptors were completed with independent samples *t*-tests or Mann–Whitney *U*-tests as appropriate. To determine the PA levels in the COV participants and any changes over time, two separate mixed-model repeated-measures analysis of variance (ANOVA) procedures examined the individual between-subject (BS) effects of sex or the persistence of symptoms on the within-subject (WS) difference in self-reported PA intensity variables at the time points and the interaction of these two accordingly to determine if the effect of time differed between subjects. Follow-up analyses were planned to determine the difference between subjects using unadjusted Mann–Whitney *U*-tests to assess differences between the sexes or the persistence of symptoms between time points for the PA intensity and total METs variables obtained from the IPAQ-SF. Spearman correlation analyses were conducted to examine relationships between variables of PA and persistent symptoms, and correlation coefficients were calculated to assess the strength and direction of these relationships. Data are reported as means ± standard deviation (SD) and median and interquartile range. Statistical significance was set at *p* < 0.05. Data were analyzed using Statistical Package for the Social Sciences software (SPSS version 29.0.0.0, IBM, New York, NY, USA) and GraphPad Prism (version 10.0.0 for Windows, GraphPad Software, Boston, MA, USA).

## 3 Results

### 3.1 Participants and baseline measurements

In total, 64 COV participants (40 ± 18 years, 45 female and 19 male participants) completed the laboratory session between February 2021 and October 2022 (8.5 ± 4.7 months after infection). The characteristics of the COV participants are reported in Table 1. Furthermore, 51 participants (80% of the COV cohort) were tested in 2021 and 13 participants (20%) in 2022. Participants identified as Caucasian (95%) or Asian (5%) and non-Hispanic (94%) or Hispanic (6%). Compared with the female participants, COV male participants were taller with greater body mass and similar BMI, lower body fat percentage, higher systolic blood pressure, and lower resting heart rates (Table 1).

**Table 1:** COV participant characteristics—comparisons between female and male participants.

Variable	Total	Female	Male	Sig. (*p*)
*n*, count (%)	64	45 (70%)	19 (30%)	—
Age (years)	40 ± 18	37 ± 17	45 ± 20	0.236
Age group, count (%)				—
Young adult (18–39 years)	36 (56%)	28 (62%)	8 (42%)	
Middle age (40–59 years)	16 (25%)	11 (25%)	5 (26%)	
Older adult (60+ years)	12 (19%)	6 (13%)	6 (32%)	
Height (cm)	170.3 ± 8.6	167.0 ± 6.1	178.3 ± 8.3	<0.001^a^
Weight (kg)	73.6 ± 15.7	70.0 ± 14.0	82.1 ± 17.0	0.004^a^
BMI	25.3 ± 4.7	25.1 ± 5.1	25.6 ± 3.9	0.485
BMI category, count				—
Underweight (<18.5)	2	2	0	
Normal weight (18.5–24.9)	29	22	7	
Overweight (25–29.9)	24	15	9	
Obese (≥30)	9	6	3	
Body fat (%)	29.3 ± 8.4	32.5 ± 7.5	21.8 ± 4.7	<0.001^a^
Systolic BP (mmHg)	122 ± 17	118 ± 15	130 ± 20	0.010^a^
Diastolic BP (mmHg)	75 ± 11	74 ± 10	76 ± 13	0.600
Resting HR (bpm)	67 ± 12	69 ± 12	61 ± 10	0.021^a^

Data are represented as mean ± standard deviation unless otherwise mentioned.

^a^ Statistically significant differences between male and female participants (sex effect, *p* < 0.05).

A subset of 43 of the 64 COV participants (67%) (46 ± 18 years, 28 female and 15 male participants) completed the online follow-up survey between May 2024 and June 2024 (51.0 ± 39.7 months, i.e., 4.25 years after infection).

Moreover, 22 of the 64 COV participants (35 ± 16 years, 16 female and 6 male participants) were matched for age, sex, and BMI with CON participants. The characteristics of the matched pairs are reported in Table 2. The matched pairs completed the laboratory session 2.0 ± 1.6 months apart between February 2021 and April 2022 and as expected, did not differ in age, sex, or BMI. In addition, there was no difference between pairs for height, body fat percentage, resting blood pressure, or resting heart rate. However, the COV participants were 2.1 kg heavier than the CON participants (Table 2). At the laboratory session, all the matched CON participants (*n* = 22) and 16 of the 22 COV participants reported being vaccinated against COVID-19. Upon completion of the follow-up survey, three of these COV individuals reported being vaccinated sometime after completing the laboratory session.

**Table 2:** Matched participant characteristics of COV and CON (*n* = 22 pairs).

Variable	COV	CON	Sig. (*p*)
*n*	22	22	—
Sex, count (%)	F, 16 (73%)	F, 16 (73%)	—
	M, 6 (27%)	M, 6 (27%)	
Age (years)	35 ± 16	36 ± 16	0.072
Age group, count (%)			—
Young adult (18–39 years)	14 (64%)	14 (64%)	
Middle age (40–59 years)	6 (27%)	6 (27%)	
Older adult (60+ years)	2 (9%)	2 (9%)	
Height (cm)	168.2 ± 6.6	168.1 ± 7.7	0.920
Weight (kg)	66.1 ± 9.1	64.0 ± 9.6	0.042^a^
BMI	23.4 ± 3.0	22.6 ± 2.6	0.114
BMI category, count			—
Underweight (<18.5)	0	1	
Normal weight (18.5–24.9)	14	15	
Overweight (25–29.9)	8	6	
Obese (≥30)	0	0	
Body fat (%)	24.7 ± 8.8	23.8 ± 7.6	0.439
Systolic blood pressure (mmHg)	121 ± 16	121 ± 16	0.848
Diastolic blood pressure (mmHg)	72 ± 12	73 ± 9	0.540
Resting heart rate (bpm)	66 ± 11	66 ± 14	0.951

Data are represented as mean ± standard deviation unless otherwise mentioned.

^a^ Statistically significant differences between pairs (*p* < 0.05).

### 3.2 COVID-19 history

The COV participants reported a wide range of symptoms affiliated with COVID-19 at three different time points including (1) during acute COVID-19 infection, (2) at the laboratory session (8.5 months on average after infection), and (3) during the follow-up survey (4.25 years on average after infection). All the COV participants reported at least one symptom during the acute infection.

**Figure 1:**
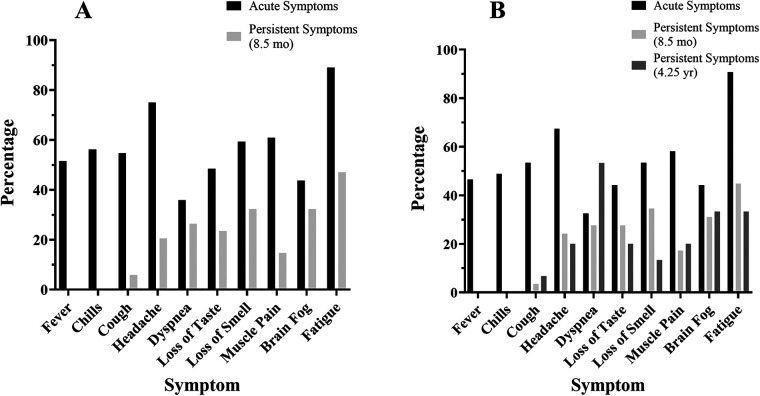
COVID-19 symptoms at the time of infection and post-infection. COVID-19 symptoms reported by the COV participants (% of participants) at each time point: at the time of acute infection (black), persistent symptoms at 8.5 months (light gray), and persistent symptoms at 4.25 years (dark gray). **(A)** Symptom prevalence reported by the 64 COV participants at the laboratory session and **(B)** symptom prevalence reported by the 43 COV participants who participated in the follow-up survey.

Of the 64 COV participants, 34 (53%) reported persistent symptoms at the laboratory session (8.5 months after infection), which was the same for female and male participants (Table 3). The most common symptom reported was fatigue as 57 of 64 COV participants (89%) reported fatigue acutely and 16 of 34 COV participants (47%) reported fatigue 8.5 months after infection when tested at the laboratory session (Figure 1).

**Table 3:** Number of COVID-19 survivors with persistent symptoms at 8.5 months and 4.25 years after infection.

Variable	*n* (%)
Laboratory session (8.5 ± 4.7 months) (*n* = 64)	
No symptoms	30 (47%)
Female	21 (47%)
Male	9 (47%)
Persistent symptoms	34 (53%)
Female	24 (53%)
Male	10 (53%)
Follow-up (51.0 ± 39.7 months, i.e., 4.25 years) (*n* = 43)	
No symptoms	28 (65%)
Female	19 (68%)
Male	9 (60%)
Persistent symptoms	15 (35%)
Female	9 (32%)
Male	6 (40%)

The percentages reported are of the total sample and for the number of female or male participants at that time interval of testing.

Of the 43 COV participants who completed the follow-up survey (4.25 years after infection), 15 (35%) reported persistent symptoms (32% of the female and 40% of the male participants) (Table 3). Furthermore, 29 of the 34 COV participants who reported persistent symptoms at the laboratory session completed the follow-up survey and 14 COV participants continued to report persistent symptoms at 4.25 years (Table 3). The most common symptom reported at the follow-up was fatigue with 5 of 15 COV participants (33%) (Figure 1).

### 3.3 Physical activity questionnaire (IPAQ-SF) over time

To understand the changes in PA in the COV participants, the IPAQ-SF asked questions about the intensity of PA before COVID-19 infection, after COVID-19 infection at 8.5 months, and at a follow-up survey 4.25 years later. PA decreased for the 64 COV participants between pre-COVID-19 infection to 8.5 months after infection. Analysis using unadjusted Wilcoxon signed-rank tests investigating the within-subject effect of time showed that all PA levels were significantly reduced with time in the COV cohort. On average, vigorous PA decreased by 75 min/week (34%, *p* < 0.001), moderate PA by 62 min/week (31%, *p* < 0.001), walking by 46 min/week (15%, *p* = 0.027), and total METs by 1,000/week (27%, *p* < 0.001). Sitting time, however, before and after COVID did not reach statistical significance although it trended in the direction of an increase of 21 min/day (8%, *p* = 0.107) (Table 4).

**Table 4:** Self-reported PA questionnaire for COV participants before and after COVID-19 infection.

PA (min/week)	Pre-COVID-19 infection		8.5 months after COVID-19 infection		4.25 years after COVID-19 infection	
	*n* = 64		*n* = 64		*n* = 43	
	Mean ± SD	Median	Mean ± SD	Median	Mean ± SD	Median
Vigorous	228 ± 202	180	153 ± 152	120	166 ± 138	135
Moderate	206 ± 174	180	144 ± 125	120	151 ± 172	120
Walking	305 ± 236	218	259 ± 177	210	285 ± 301	150
Total METs	3,656 ± 1,994	3,093	2,656 ± 1,483	2,314	2,875 ± 1,960	2,394
Sitting (min/day)	*n* = 63		*n* = 63		*n* = 43	
	276 ± 127	300	297 ± 120	300	293 ± 139	300

Data are represented as mean ± standard deviation and median.

**Figure 2:**
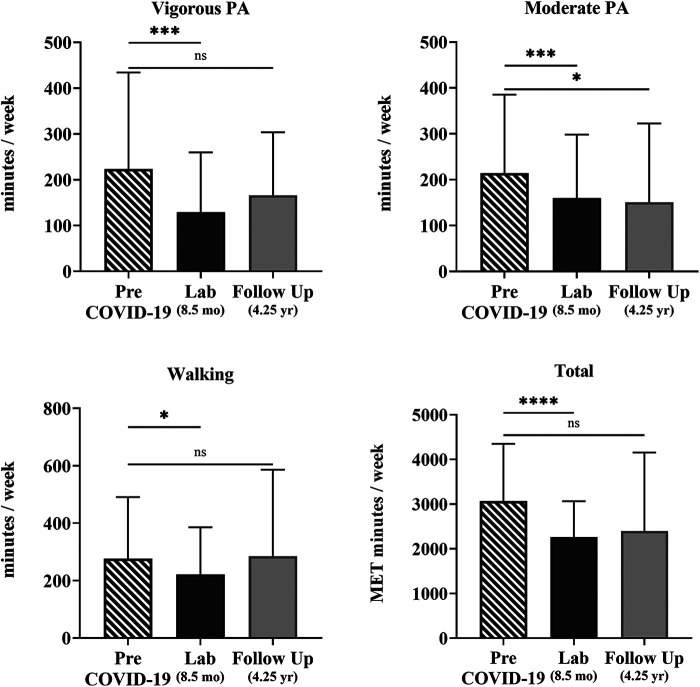
Self-reported physical activity over time of the COV participants. Shown are vigorous and moderate PA, walking, and total METs min/week quantified from the self-reported IPAQ-SF questionnaire for 43 COV participants. Shown are the mean ± SD for all graphs except for total METs, which is presented as a median and interquartile range for each time point: pre-COVID-19 infection (striped), 8.5 months later at the laboratory session (black), and 4.25 years later at the online follow-up survey (gray). The solid lines show the unadjusted effect between individual time points with statistical significance; ns = *p* > 0.05; *p ≤ 0.05; **p ≤ 0.01; ***p ≤ 0.001; ****p ≤ 0.0001.

For the 43 COV participants who completed the follow-up survey 4.25 years after infection, PA levels remained lower than pre-COVID-19 infection reports. On average, moderate PA was 63 min/week (29%, *p* = 0.048) lower at 4.25 years than pre-COVID-19 infection reports (Table 4, Figure 2). However, analysis using unadjusted Wilcoxon signed-rank tests comparing pre-COVID-19 infection reports with PA levels reported at the follow-up showed that the PA levels and sitting time were not significantly reduced.

### 3.4 Sex differences in physical activity (IPAQ-SF) over time

**Figure 3:**
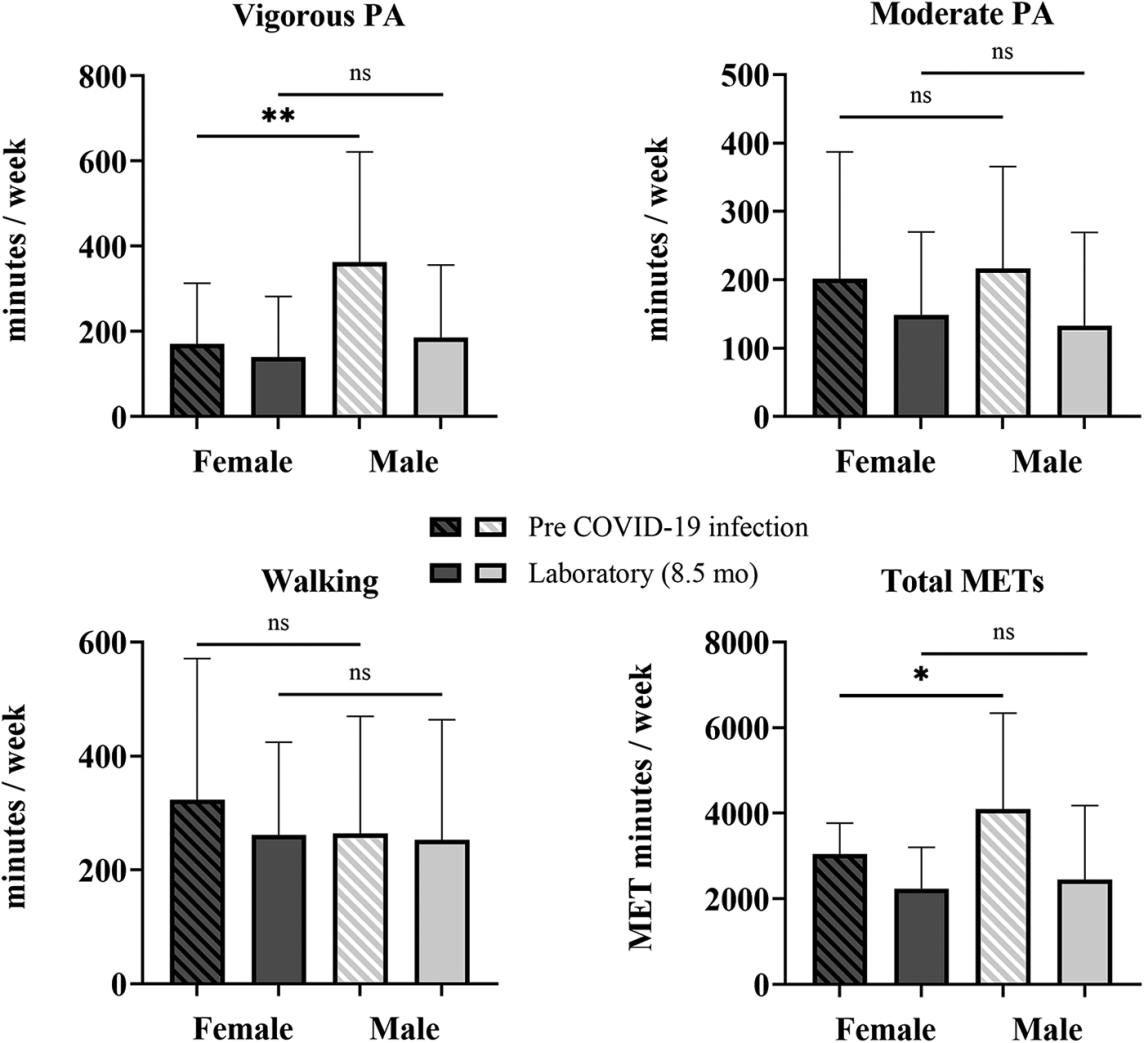
Sex differences in self-reported physical activity. Shown are vigorous and moderate PA, walking, and total METS quantified from the self-reported IPAQ-SF questionnaire for COV participants: 45 female and 19 male participants. Shown are the mean ± SD for all graphs except for total METs, which is presented as a median and interquartile range, where female participants are represented by the darker bars and male participants are represented by the lighter bars at pre-COVID-19 infection (striped) and 8.5 months later at the laboratory session (solid). The solid lines above show the preliminary unadjusted between-subject effect of sex with statistical significance; ns = *p* > 0.05; * *p* ≤ 0.05; **p ≤ 0.01.

Changes in PA from before COVID-19 infection to the time of testing in the laboratory session (8.5 ± 4.7 months after infection) for female (*n* = 45) and male (*n* = 19) participants were determined with a mixed-model repeated-measures ANOVA (Supplementary Table 2). There was a significant interaction of time × sex for vigorous PA [F (1,62) = 14.726, *p* < 0.001, *η*^2^ = 0.192] and total METs [F (1,62) = 7.097, *p* = 0.010, *η*^2^ = 0.103], indicating that the change between pre-COVID-19 infection and 8.5 months post-COVID-19 infection in both vigorous PA and total METs per week differed by sex. Mann–Whitney *U*-tests revealed that COV male participants had higher levels of vigorous PA by 192 min/week (72%, *p* = 0.002) and total METs by 1,399/week (36%, *p* = 0.017) than female participants (Figure 3). At 8.5 months post-infection, there were no differences in PA levels between female and male participants. However, sitting time was greater in male than female participants by 66 min/day (21%, *p* = 0.039).

### 3.5 Physical activity (IPAQ-SF) over time with and without persistent symptoms

**Figure 4:**
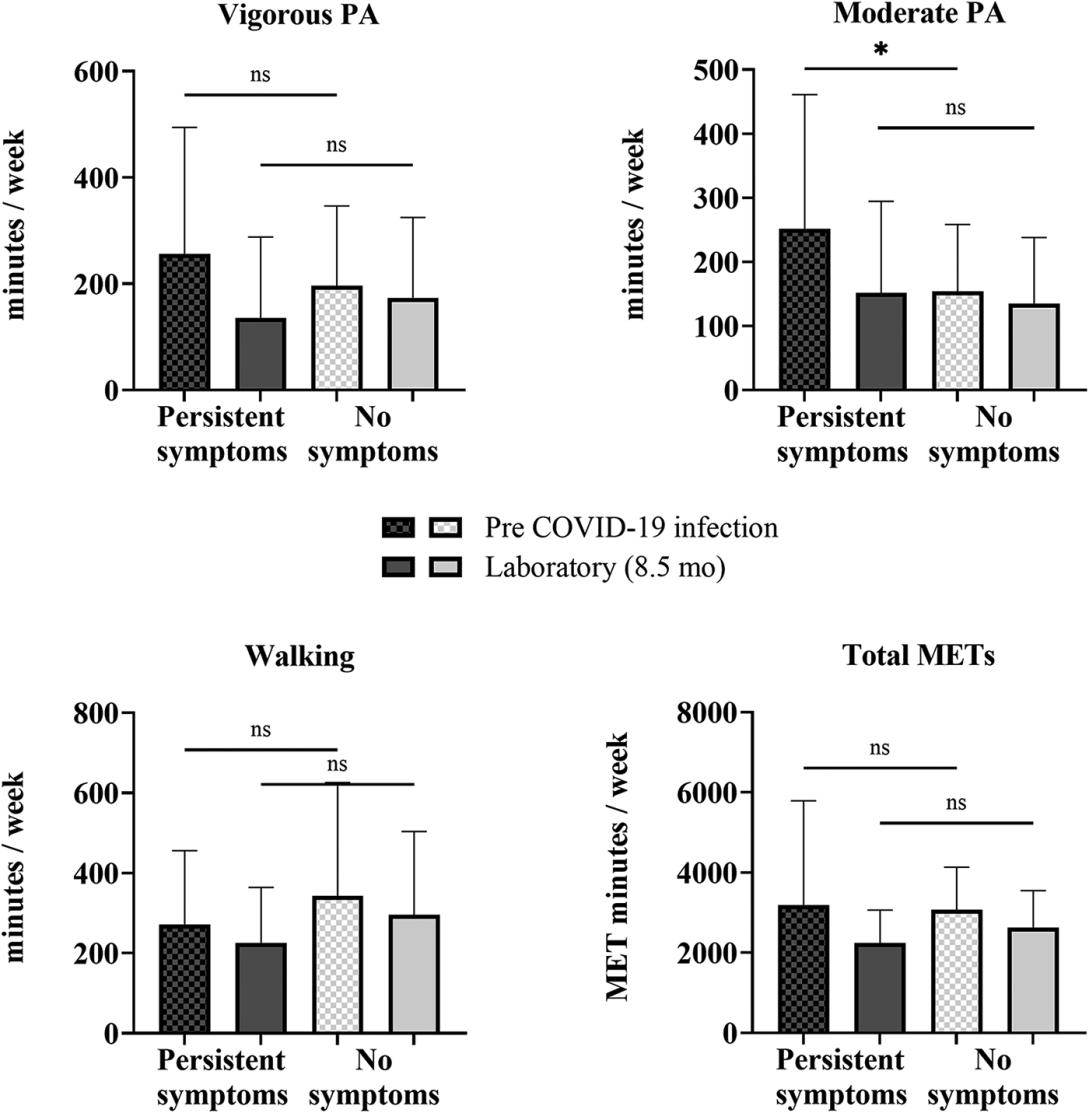
Self-reported physical activity between COV participants with and without persistent symptoms. Shown are vigorous and moderate PA, walking, and total METS quantified from the self-reported IPAQ-SF questionnaire for COV participants: 34 with persistent symptoms at 8.5 months and 30 with no symptoms at 8.5 months. Shown are the mean ± SD for all graphs except for total METs, which is presented as a median and interquartile range, where the COV participants with persistent symptoms are represented by the darker bars and COV participants without symptoms are represented by the lighter bars at pre-COVID-19 infection (checkered) and 8.5 months later at the laboratory session (solid). The solid lines above show the preliminary unadjusted between-subject effect of sex with statistical significance; ns = *p* > 0.05; *p ≤ 0.05.

Changes in PA from before COVID-19 infection to the time of testing in the laboratory session (8.5 ± 4.7 months after infection) for the COV participants who reported persistent symptoms (*n* = 34) at 8.5 months and the COV participants who reported no symptoms (*n* = 30) at 8.5 months were determined with a mixed-model repeated-measures ANOVA (Supplementary Table 3). There was a significant interaction of time × persistence of symptoms for vigorous PA [F (1,62) = 7.286, *p* = 0.009, *η*^2^ = 0.105], moderate PA [F (1,62) = 5.045, *p* = 0.028, *η*^2^ = 0.075], and total METs [F (1,62) = 8.513, *p* = 0.005, *η*^2^ = 0.121], indicating that the change between pre-COVID-19 infection and the laboratory session in these self-reported IPAQ-SF variables differed by the persistence of symptoms (persistent symptoms vs. no symptoms). Mann–Whitney *U***-**tests revealed that the COV participants who reported persistent symptoms at 8.5 months after infection (*n* = 34), in contrast to expectations, reported higher moderate PA levels pre-COVID-19 infection than those who reported no symptoms (*n* = 30) at 8.5 months after infection (Figure 4). Prior to COVID-19 infection, those who reported persistent symptoms at 8.5 months had higher levels of moderate PA (by 98 min/week) than those who did not report symptoms (48%, *p* = 0.047). At 8.5 months post-infection, there were no differences in PA levels between those with persistent symptoms at 8.5 months and those without any symptoms at 8.5 months (Figure 4).

### 3.6 Accelerometry to measure physical activity

Of the 64 COV participants, 56 (87.5%) had valid accelerometer data when collected following the laboratory session (8.5 months after infection). Wear times of 5–7 days were included (6.4 ± 0.77 days) and a minimum of 600 min/day was included in the analysis (821 ± 72 min average wear time per day). The COV cohort was divided based on the presence of persistent symptoms (*n* = 29) and those who did not report symptoms (*n* = 27) at the laboratory session (8.5 months after infection). There were no differences in sedentary time per day (persistent symptoms 510 ± 66 min, no symptoms 504 ± 55 min, *p* = 0.717) or light activity (persistent symptoms 254 ± 76 min, no symptoms 290 ± 63 min, *p* = 0.063) between the groups. However, there were higher levels of activity and greater step counts in the COV participants who reported no persistent symptoms (MVPA: persistent symptoms 37 ± 21 min, no symptoms 49 ± 18 min, 28%, *p* = 0.014; steps/day: persistent symptoms 7,915 ± 3,148 steps, no symptoms 9,540 ± 2,286 steps, 19%, *p* = 0.014).

### 3.7 Accelerometry comparisons between COV and matched CON participants

To address the hypothesis that PA would be lower in the COV participants (*n* = 22) compared with matched CON participants (*n* = 22), PA from accelerometry was compared at 8.5 months after infection (time of testing of the laboratory session). Participant wear times for days worn (COV 6.3 ± 1.0 days, CON 6.6 ± 0.58 days) and minutes/day worn (COV 805 ± 62 min, CON 835 ± 79 min average wear time per day) are provided. There were no differences in activity levels between the COV and CON cohorts for light activity (COV 257 ± 69 min, CON 262 ± 57 min, *p* = 0.829), MVPA (COV 40 ± 23 min, CON 45 ± 25 min, *p* = 0.455), or steps per day (COV 8,768 ± 3,493 steps, CON 9,514 ± 3,774 steps, *p* = 0.367). In addition, there were no differences between COV and CON participants in sedentary time per day (COV 507 ± 64 min, CON 528 ± 60 min, *p* = 0.185).

**Figure 5:**
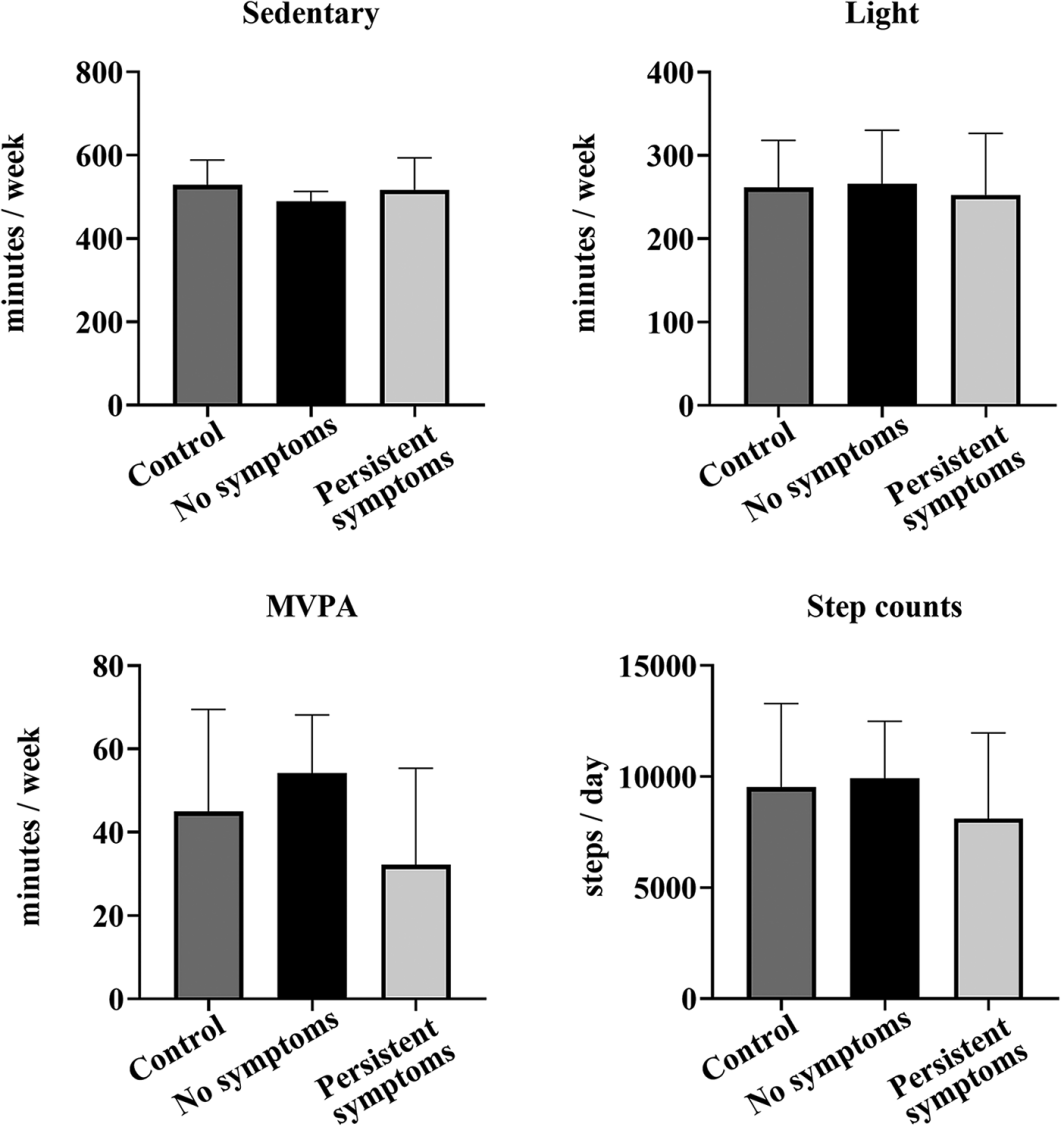
Comparison of accelerometry between the COV and CON participants. Shown are sedentary, light PA, MVPA, and step counts per day measured by accelerometry for 22 CON participants (dark gray), 8 COV participants without any symptoms at the laboratory session (black) (8.5 months after infection) and 14 COV participants with persistent symptoms at the laboratory session (light gray) (8.5 months after infection). Shown are the mean ± SD for all graphs.

The COV cohort was divided based on the presence of persistent symptoms (*n* = 14) and those who did not report symptoms (*n* = 8) at the laboratory session (8.5 months after infection), and matched with CON participants (*n* = 22) (Figure 5). One-way ANOVA analysis showed there were no differences between the groups for light activity: [F (2,43) = 0.134, *p* = 0.875], MVPA [F (2,43) = 2.645, *p* = 0.083], sedentary time/day [F (2,43) = 1.167, *p* = 0.322], or steps/day [F (2,43) = 0.864, *p* = 0.429]. However, when separate analyses were completed in the COV cohort to compare those with persistent symptoms (*n* = 14) and those who reported no symptoms (*n* = 8) at the laboratory session, MVPA was lower in those with persistent symptoms by 22 min/week (51%, *p* = 0.006). Interestingly, when the average MVPA/day completed was estimated per week by multiplying by 7 days, all eight (100%) of the COV participants who reported no symptoms met the PA guidelines of at least 150 min/week at 8.5 months. In contrast, nine of the COV participants (64%) with persistent symptoms met the guidelines.

### 3.8 Comparisons between measures of physical activity

Self-reported PA from the IPAQ-SF was also compared between COV (*n* = 22) and CON (*n* = 22) participants when captured at 8.5 months post-COVID-19 infection. There were no differences in self-reported activity levels or sitting time between the COV and CON cohorts. Comparisons were made between accelerometry and self-reported PA levels in COV and CON participants. The IPAQ-SF calculates the average minutes per week of MVPA and the accelerometer analysis calculates the average MVPA per day. To compare these variables, the IPAQ-SF average minutes per week of MVPA was divided by 7 days as an estimate to make a comparison with the average MVPA per day. Self-reported daily MVPA was comparable to daily MVPA measured via accelerometry (COV: self-reported 41 ± 28 min, accelerometer 40 ± 23 min, *p* = 0.894; CON: self-reported 55 ± 39 min, accelerometer 45 ± 25 min, *p* = 0.196).

### 3.9 Associations between measures of physical activity and persistent symptoms

Correlation analyses were conducted to assess the relationship between the number of symptoms reported by the COV participants (*n* = 64) during the acute infection and at the laboratory session (8.5 months after infection) and self-reported PA measures (IPAQ-SF). There were no significant correlations between pre-COVID-19 infection PA levels and the number of acute symptoms or with the number of persistent symptoms at 8.5 months, suggesting that pre-COVID-19 infection PA levels were not protective for acute COVID-19 or long COVID symptoms. However, associations were found at 8.5 months post-infection between vigorous PA (r_s_ = −0.250, *p* = 0.046) and total METs (r_s_ = −0.264, *p* = 0.035) with the number of persistent symptoms, suggesting that lower PA levels are associated with more persistent reported symptoms when assessed at 8.5 months. Additional analyses found no association between the number of symptoms reported by the COV participants (*n* = 43) at 4.25 years after infection (online follow-up) and any of the IPAQ-SF variables.

Furthermore, in the COV participants (*n* = 56), there were negative associations between the number of symptoms and MVPA (r_s_ = −0.283, *p* = 0.034) and steps per day (r_s_ = −0.340, *p* = 0.010) measured with accelerometry, providing further evidence to suggest that lower PA levels are associated with more persistent symptoms at 8.5 months post-infection.

## 4 Discussion

The aims of this study were to determine the impact of the COVID-19 pandemic on PA levels and the influence of PA on acute and persistent symptoms of non-hospitalized COVID-19 survivors. We hypothesized that (1) PA levels would decrease following acute COVID-19 infection but recover over time, (2) PA levels would be lower in COV participants than matched CON participants, and (3) those who reported higher PA levels prior to infection would have the greatest protection against long COVID. We found that our sample of non-hospitalized COVID-19 survivors experienced a decline in reported PA levels with acute infection and at 8.5 months on average after infection. However, when reassessed 4.25 years on average after infection, PA levels were largely restored to pre-pandemic levels. The decline in PA at 8.5 months after infection for the COV participants was also experienced in the matched controls who were tested at the same time during the pandemic. This suggests that the stay-in-place orders and the pandemic itself resulted in a reduction in activity levels for many people whether or not they had been infected with COVID-19. Interestingly, 53% of our COV cohort experienced at least one long COVID symptom (the most common was fatigue) at 8.5 months post-infection, and 35% of the sample did at the 4.25-year follow-up. Contrary to our hypothesis, those who were most active prior to COVID-19 infection were not protected against long COVID. We found that COVID-19 infection and persistent symptoms impacted people who participated in high-intensity PA prior to infection (self-reported and accelerometry). Finally, male participants and those with persistent symptoms at the laboratory session (8.5 months post-infection) had greater reductions in PA levels following COVID-19 infection than female participants and those reporting no persistent symptoms.

### 4.1 Persistent symptoms of COVID-19 were prevalent in non-hospitalized COV participants

Self-reported symptoms for the acute infection phase showed that, as expected, the COV participants experienced fatigue, headache, muscle pain, loss of smell, chills, cough, and fever. However, 8.5 months on average after acute infection, 53% of the COV non-hospitalized participants reported persistent symptoms. The most common symptoms reported at this time were fatigue, brain fog, loss of smell, and dyspnea. In addition, in a follow-up survey 4.25 years after acute infection, 35% of the participants reported persistent symptoms that included dyspnea, fatigue, and brain fog. These findings are consistent with the Centers for Disease Control and Prevention for common symptoms acutely and persistently (3, 7). In an international online survey of 3,762 participants with COVID-19, 65% of respondents experienced persistent symptoms for at least 6 months and the most common persistent symptoms were fatigue, cognitive dysfunction, headaches, and memory issues (48). While few studies have followed patients more than 1 year after infection and none have done so in non-hospitalized COVID-19 survivors, one study of previously hospitalized patients with long COVID reported that in 199 patients, the most common symptoms at 26 months after infection were fatigue, shortness of breath, and loss of smell and taste (49). Our findings indicate that even in non-hospitalized COVID-19 survivors, over 1/3 of the cohort (35%) reported experiencing persistent symptoms up to and over 4 years post-infection.

### 4.2 Physical activity levels declined during the pandemic

Overall, self-reported PA declined from pre-COVID-19 infection levels to 8.5 months following infection with COVID-19 with the greatest declines in higher PA intensities (34% decrease in vigorous intensity PA and 31% decrease in moderate-intensity PA). When PA was assessed again 4.25 years following infection in the same individuals, PA levels remained lower than pre-COVID-19 infection reports where vigorous PA was 26% lower and moderate PA was 29% lower. These findings were similar to Dunton et al. who reported a 35% decrease and 46% decrease in vigorous and moderate PA, respectively, measured with the IPAQ-SF from pre-COVID-19 pandemic (February 2020) to 2 months later (April 2020) (44). Other studies reported that the most active individuals had the highest decrease in vigorous PA when compared with less active individuals (50, 51). Our findings align with these studies, despite our participants being non-hospitalized and generally more active overall.

While the self-reported questionnaire data indicated activity levels declined in the COV participants at 8.5 months, we also showed no differences in activity levels between the matched controls and COVID-19 survivors in the self-report questionnaire and accelerometry data. This important finding suggests that the decline in activity levels was global and due to the pandemic and not exclusive to prior COVID-19 infection. We further examined this with the accelerometry data, comparing the COV participants with persistent symptoms to those without symptoms and to CON participants: the lack of differences in activity between the groups confirms that the pandemic had a large effect by lowering activity levels population-wide.

### 4.3 Effect of persistent symptoms on activity levels

The COV participants who reported persistent symptoms had reduced levels of PA. A comparison of accelerometry data between COV participants with persistent symptoms and those without showed that those with persistent symptoms engaged in less MVPA. The Physical Activity Guidelines for Americans recommend that healthy adults (18–65 years) should participate in at least 150 min of moderate-intensity physical activity each week (30). Despite the health benefits, ∼47% American adults do not meet these exercise guidelines (30). From the measured accelerometer data in 22 COV participants and 22 matched CON participants, we found that 77% of our sample met PA guidelines, indicating overall that our sample was highly active. We also showed that for activity assessed with accelerometry, 100% of the COV participants who reported no symptoms met the PA guidelines whereas only 64% of those with persistent symptoms met them. This finding further shows that even in a highly active group, persistent symptoms had an impact, resulting in lower PA levels at even 8.5 months after infection.

In addition, in contrast to expectations, people with persistent symptoms at 8.5 months after infection self-reported higher moderate PA levels pre-COVID-19 infection than those who reported no symptoms at 8.5 months after infection. This was surprising because we expected that people who were the most active prior to COVID-19 infection would be protected against long COVID. Previous studies in the literature on acute COVID-19 infection and the risk of hospitalization due to COVID-19 have shown that inactivity was the strongest risk factor of hospitalization, ultimately suggesting that PA may be protective against acute infection (21, 31, 32). Activity levels prior to infection, therefore, are not protective against long COVID symptoms, suggesting activity-related mechanisms that protect against other chronic diseases are not relevant to long COVID.

### 4.4 Limitations

There are several limitations in this study. First, both groups of participants in our study were highly active and may not be representative of the general American population. Our participants were primarily Caucasian and non-Hispanic, yet Black and Hispanic populations experienced disproportionately higher rates of COVID-19 infection and related mortality (52). In addition, the COVID-19 pandemic impacted the most disadvantaged populations concerning the social determinants of health, which were not explored in this study (53). Further, there is no measurable clinical tool currently available to diagnose long COVID and therefore self-reported symptoms were reported. There is a need to establish a quantifiable tool that considers the prevalence and duration of long COVID (54). In addition, there may have been a self-selection bias because COVID-19 survivors with persistent symptoms were likely more inclined to participate in the laboratory session and the subsequent follow-up survey. Finally, self-reported measures of activity levels (IPAQ-SF) may be higher compared with data from accelerometry. In addition, the use of retrospective self-reported measures to assess activity levels prior to COVID-19 infection may have introduced reporting biases with the IPAQ-SF.

## 5 Conclusion

This study is the first to our knowledge to study and understand physical activity engagement and persistent symptoms in non-hospitalized COVID-19 survivors roughly 4 years after infection. We showed that activity levels were reduced likely as a result of the pandemic even 8.5 months after infection in the COV participants and healthy matched controls who had no known prior COVID-19 infection. However, even those who reported high levels of activity prior to COVID-19 infection experienced persistent symptoms and hence activity was not protective against long COVID. Fatigue was notably the most common symptom across all time points and even at just over 4 years post-infection. Health professionals and researchers should not overlook COVID-19 survivors who were not hospitalized for their infection of COVID-19 as these individuals continue to experience persistent symptoms that impact engagement in activity. Finally, male participants and people with persistent symptoms of long COVID had the greatest reduction in activity levels. Conducting longitudinal studies on non-hospitalized COVID-19 survivors who represent the majority of those affected by COVID-19 infection will be important to understand the lasting impact of activity levels in more diverse populations.

## Data Availability

The raw data supporting the conclusions of this article will be made available by the authors, without undue reservation.
