# Supplementary material for: COVID-19 and the impact of physical activity on persistent symptoms
Source: Front Sports Act Living. 2025 Apr 24;7:1560023. doi: 10.3389/fspor.2025.1560023 (PMC12058785; doi:10.3389/fspor.2025.1560023)
Supplement: Supplementary file 1 [file Table1.docx]

**Supplemental Table 1. COVID-19 Symptom List**

| Cough (new onset or worsening of chronic cough) |
| --- |
| Shortness of breath |
| Fever (temperature > 100 deg Fahrenheit) |
| Chills |
| Sore throat |
| Runny nose |
| Muscle pain |
| Headache |
| Loss of taste |
| Loss of smell |
| COVID toes (swelling and discoloration of the toes) |
| Chest pain |
| Body rash |
| Hair loss |
| Anemia |
| Joint pain |
| Brain fog (difficulty with thinking, concentration, and memory) |
| Fatigue |
| Depression |
| Anxiety |
| Changes in mood |
| Heart palpitations (fast-beating or pounding heart) |
| Other* |

**Supplemental Table 1.** COV participants were provided this symptom list

*Open-ended question to allow free response
