# Supplementary material for: COVID-19 and the impact of physical activity on persistent symptoms
Source: Front Sports Act Living. 2025 Apr 24;7:1560023. doi: 10.3389/fspor.2025.1560023 (PMC12058785; doi:10.3389/fspor.2025.1560023)
Supplement: Supplementary file 2 [file Table2.docx]

**Supplemental Table 2. Physical Activity Levels (Self-reported PA, IPAQ-SF) in Female and Male COV Participants Across Time (Pre Covid vs 8.5 Months Post Infection).**

| **Variable**  **(n=64)** | **Vigorous (min/week)** | | | | **Moderate (min/week)** | | | | **Walking (min/week)** | | | | **Total METs (min/week)** | | | |
| --- | --- | --- | --- | --- | --- | --- | --- | --- | --- | --- | --- | --- | --- | --- | --- | --- |
|  | df | F | p | η2 | df | F | p | η2 | df | F | p | η2 | df | F | p | η2 |
| **Time (WS)** | 1 | 30.378 | ***<0.001*** | 0.329 | 1 | 11.121 | ***0.001*** | 0.152 | 1 | 2.991 | 0.089 | 0.046 | 1 | 34.035 | ***<0.001*** | 0.354 |
| **Sex (BS)** | 1 | 8.055 | ***0.006*** | 0.115 | 1 | 0.000 | 0.987 | 0.000 | 1 | 0.408 | 0.525 | 0.007 | 1 | 4.033 | ***0.049*** | 0.061 |
| **Time (WS) x Sex (BS)** | 1 | 14.726 | ***<0.001*** | 0.192 | 1 | 0.591 | 0.445 | 0.009 | 1 | 1.423 | 0.237 | 0.022 | 1 | 7.097 | ***0.010*** | 0.103 |

**Supplemental Table 2**. Mixed Model Repeated-Measures ANOVA - Examining the Within-Subjects Effects of Time (pre COVID-19 infection vs. 8.5 months after infection) on Self-reported PA (IPAQ-SF) by the Between-Subject Effects of Sex. n = 64 for all PA variables. η2 = effect size (partial eta squared). WS = within-subject effect. BS = between-subject effect. Self-reported minutes of vigorous and moderate PA, walking, and total METs per week were assessed by the IPAQ-SF across two different time points including (1) prior to COVID-19 infection and (2) at the laboratory session (8.5 months after infection). Sex (female (n=45) vs. male (n=19)).
