# Supplementary material for: COVID-19 and the impact of physical activity on persistent symptoms
Source: Front Sports Act Living. 2025 Apr 24;7:1560023. doi: 10.3389/fspor.2025.1560023 (PMC12058785; doi:10.3389/fspor.2025.1560023)
Supplement: Supplementary file 3 [file Table3.docx]

**Supplemental Table 3. Physical Activity Levels (Self-reported PA, IPAQ-SF) in COV Participants with and Without Persistent Symptoms Across Time (Pre Covid vs 8.5 Months Post Infection).**

| **Variable**  **(n=64)** | **Vigorous (min/week)** | | | | **Moderate (min/week)** | | | | **Walking (min/week)** | | | | **Total METs (min/week)** | | | |
| --- | --- | --- | --- | --- | --- | --- | --- | --- | --- | --- | --- | --- | --- | --- | --- | --- |
|  | df | F | p | η2 | df | F | p | η2 | df | F | p | η2 | df | F | p | η2 |
| **Time (WS)** | 1 | 15.475 | ***<0.001*** | 0.200 | 1 | 10.734 | ***0.002*** | 0.148 | 1 | 5.716 | ***0.020*** | 0.084 | 1 | 25.609 | ***<0.001*** | 0.292 |
| **Persistence of Symptoms (BS)** | 1 | 0.076 | 0.783 | 0.001 | 1 | 3.112 | 0.083 | 0.048 | 1 | 2.157 | 0.147 | 0.034 | 1 | 0.048 | 0.827 | 0.001 |
| **Time (WS) x Persistence of Symptoms (BS)** | 1 | 7.286 | ***0.009*** | 0.105 | 1 | 5.045 | ***0.028*** | 0.075 | 1 | 0.000 | 0.998 | 0.000 | 1 | 8.513 | ***0.005*** | 0.121 |

**Supplemental Table 3**. Mixed Model Repeated-Measures ANOVA - Examining the Within-Subjects Effects of Time (pre COVID-19 infection vs. 8.5 months after infection) on Self-reported PA (IPAQ-SF) by the Between-Subject Effects of Persistence of Symptoms.

n = 64 for all PA variables. η2 = effect size (partial eta squared). WS = within-subject effect. BS = between-subject effect. Self-reported minutes of vigorous and moderate PA, walking, and total METs per week were assessed by the IPAQ-SF across two different time points including (1) prior to COVID-19 infection and (2) at the laboratory session (8.5 months after infection). Persistence of symptoms (no symptoms (n=30) vs. persistent symptoms (n=34)).
